# Biodiversity and Habitats of Polar Region Polyhydroxyalkanoic Acid-Producing Bacteria: Bioprospection by Popular Screening Methods

**DOI:** 10.3390/genes11080873

**Published:** 2020-07-31

**Authors:** Małgorzata Marta Rogala, Jan Gawor, Robert Gromadka, Magdalena Kowalczyk, Jakub Grzesiak

**Affiliations:** 1 Department of Antarctic Biology, Institute of Biochemistry and Biophysics, Polish Academy of Sciences Pawińskiego 5A, 02-106 Warszawa, Poland; m.wroblewska@ibb.waw.pl; 2 Laboratory of DNA Sequencing and Oligonucleotide Synthesis, Institute of Biochemistry and Biophysics, Polish Academy of Sciences, Pawińskiego 5A, 02-106 Warszawa, Poland; gaworj@ibb.waw.pl (J.G.); robert@ibb.waw.pl (R.G.); 3 Department of Microbial Biochemistry, Institute of Biochemistry and Biophysics, Polish Academy of Sciences Pawińskiego 5A, 02-106 Warszawa, Poland; mk@ibb.waw.pl

**Keywords:** psychrophiles, Nile Red, *Comamonadaceae*, glacier, feast/famine regime

## Abstract

Polyhydroxyalkanoates (PHAs), the intracellular polymers produced by various microorganisms as carbon and energy storage, are of great technological potential as biodegradable versions of common plastics. PHA-producing microbes are therefore in great demand and a plethora of different environments, especially extreme habitats, have been probed for the presence of PHA-accumulators. However, the polar region has been neglected in this regard, probably due to the low accessibility of the sampling material and unusual cultivation regime. Here, we present the results of a screening procedure involving 200 bacterial strains isolated from 25 habitats of both polar regions. Agar-based tests, microscopy, and genetic methods were conducted to elucidate the biodiversity and potential of polar-region PHA-accumulators. Microscopic observation of Nile Red stained cells proved to be the most reliable screening method as it allowed to confirm the characteristic bright orange glow of the Nile Red–PHA complex as well as the typical morphology of the PHA inclusions. Psychrophilic PHA-producers belonged mostly to the *Comamonadaceae* family (Betaproteobacteria) although actinobacterial PHA synthesizers of the families, *Microbacteriaceae* and *Micrococcaceae* also featured prominently. Glacial and postglacial habitats as well as developed polar region soils, were evaluated as promising for PHA-producer bioprospection. This study highlights the importance of psychrophiles as biodiverse and potent polyhydroxyalkanoate sources for scientific and application-aimed research.

## 1. Introduction

Polyhydroxyalkanoates (PHA) are a group of intracellular polymers synthesized by a variety of prokaryotic microorganisms. Their primary function is that of a carbon and energy storage to be used in starvation periods. They are produced from the excess carbon present in the cells’ environment and consist of polyesters of hydroxyalkanoic acids with chains of varying lengths. Key enzymes conducting the polymerization process are the PHA synthases encoded by the *phaC* gene. Currently, there are four classes of this enzyme discovered and described. Amino acid sequence overlap between enzymes representing different classes is moderate at best, albeit their products can be divided into short side chain length PHAs (scl-PHAs) produced mainly by the actions of the I, III and IV class synthases and medium side chain length PHAs (mcl-PHAs) produced mainly by the class II synthases harbored *inter alia* by the *Pseudomonas* genus [1,2].

Polyhydroxyalkanoic acids are of great technological potential because they are seen as a possible petroleum-based plastic substitute: biodegradable, biocompatible, and derived from biowaste products [3,4]. Therefore, PHA producing microbes are highly sought after in terms of quality and quantity of the synthesized polymer [5]. Screening procedures involve mainly the detection of the PHA granule themselves and/or the detection of genes coding the PHA synthesis pathway enzymes [6]. Staining of the granules with lipophilic dyes is the most popular of the methods, with the fluorescent Nile Red stain being frequently used to differentiate PHA positive bacterial colonies on a Petri dish or to visualize granules within bacterial cells using an adequately equipped microscope. The PHA positive strains display a bright orange glow when irradiated with UV (330–380 nm) or green light (510–560 nm) [7,8]. Genetic screening involves mainly the PCR based amplification of a fragment of the *phaC* gene using degenerate primers [9].

PHA producing bacteria can be found in a variety of environments, mainly those that experience periodic nutrient limitations, as the granule-stored carbon helps to cope with starvation issues [3]. Studies conducted on the survival of PHA-containing bacterial cells reveal the involvement of these polymers in cell recovery from a number of other abiotic stressors [10]. Therefore, habitats, where the intensity of physical and chemical factors change frequently, have the potency to enrich the microbial community in most resilient cells, *inter alia* in PHA producers [11]. Polar region habitats seem in this regard to be a very promising source of novel PHA synthesizing bacterial strains [12,13]. Polar and circumpolar regions experience a severe case of seasonal nutrient inputs caused by the occurrence of the yearly polar day/night phenomenon. Furthermore, several other factors (not only low temperatures) such as water activity shifts, freeze-thaw cycles, intense UV radiation, free radical formation, and sudden pH changes also contribute to the harshness of this environment [14]. Therefore, prospecting for polar region cold-loving bacterial PHA producers might prove very fruitful. Such endeavors have to date been undertaken only in a limited respect, presumably due to the low accessibility of the sampling material and unusual cultivation regime [15].

In this study, we present a screening of 200 bacterial isolates derived from 25 different habitats all located in polar regions of both the Arctic and Antarctica done by popular testing methods. The aim of these analyses was to evaluate the actual potential of those microbes to produce PHAs and additionally to find the most accurate method to do so. Our hypothesis states that polar region habitats harbor a phylogenetically and metabolically diverse cultivable bacteria communities capable of PHA production at low temperatures.

## 2. Materials and Methods

### 2.1. Strain Cultivation

Bacterial strains used in this study (see Table 1 for details) are part of the Central Collection of Strains of the Institute of Biochemistry and Biophysics Polish Academy of Sciences (http://kolekcja.ibb.waw.pl/) described in the “Arctic and Antarctic psychrophilic database” section. The strains are stored as glycerol stocks at −80 °C. They were revived by streaking aliquots of the stocks on an appropriate medium. For the saltwater isolates, R3A medium was used containing (g/L): peptone—1, tryptone—1, yeast extract—1, beef extract—1, glucose—1, K_2_HPO_4_—1, NaH_2_PO_4_—0.5, MgSO_4_—0.1, prepared with artificial seawater (g/L): NaCl—27.5, MgCl_2_·6H_2_O—5.38, MgSO_4_·7H_2_O—6.78, KCl—0.72, NaHCO_3_—0.2, CaCl_2_·2H_2_O—1.4. For all other strains, the R3A medium (agar and broth) was prepared with distilled water. The plates were incubated at 15 °C in the dark (Liebher Thermostat Cabinet) for a period of 3 weeks. Strains were checked for purity by repeated transfer to fresh agar plates. Single colonies were picked and transferred to test tubes with 3 mL of cool, sterile R3A broth and incubated for 1 week to attain a saturated bacterial culture. The submerged cultures were agitated by briefly vortexing once a day. The typical OD was estimated to have been 0.7. The cultures were the basis for further research.

### 2.2. Agar-Based Screening

The R3A agar plates for PHA screening were prepared according to [7]. Stock solutions of Nile Blue A and Nile Red dyes were prepared in DMSO and added to the agar *post* autoclaving to give a final concentration of 0.5 µg dye per mL of medium. Strains were drop plated (5 µL) from the active culture onto the plates, with controls on R3A agar without the dyes. After 1 week of incubation at 15 °C, the plates were exposed to ultraviolet light (312 nm) and the drop-spots were examined for the bright orange fluorescence indicative for PHA presence. Those exhibiting the glow were scored as positive.

### 2.3. Gene-Based Analysis

For DNA extraction from bacterial cells, 100 µL of the liquid culture was centrifuged at 12,000 rpm for 3 min in a 2 mL Eppendorf type tube and the pellet was suspended in 200 µL of sterile MiliQ water and approx. ten milligrams of Chelex100 resin and sharp garnet sand were added. The suspension was further amended with 1.5 µL of lysozyme solution (10 mg/mL) and incubated at 37 °C for 2.5 h. Then 10 µL of a 10% SDS solution was added to the suspension and the tubes were placed in a Qiagen Retsch TissueLyser II for 5 min at 39 Hz–1800 oscillation per min. The tubes were centrifuged briefly, amended with 1 µL proteinase K solution and incubated at 55 °C for 1 h. After centrifugation at 12,000 rpm for 3 min, the DNA in the supernatant was purified using the Clean-up Concentrator kit (A&A Biotechnology) according to the manufacturer’s protocol. The yield and purity of the extracted DNA were checked in a NanoPhotometer^®^ NP80 (Implen).

Amplification of the 16S rRNA gene fragment was performed using universal primers 27F and 1492R [16]. PCR conditions were as follows: 1 min of 95 °C initial denaturation followed by 30 cycles of 95 °C for 15 s, 55 °C annealing for 15 s and elongation 72 °C for 1 min and 30 s, using DreamTaq polymerase (Thermo Scientific-Fermentas). Obtained PCR products (~1500 bp for 16S rRNA gene fragment) were checked on 0.8% agarose gel and purified using the Clean-up Concentrator kit (A&A Biotechnology). The 16S rRNA gene amplicons were sequenced with PCR forward primer (27F) with the use of BigDye Terminator v.3.1 chemistry and ABI3730xl genetic analyzer at the DNA Sequencing Laboratory (Institute of Biochemistry and Biophysics PAS).

Amplification of the *phaC* gene fragment was performed using primers G-D (5′GTGCCGCCSYRSATCAACAAGT3′) and G-1R (5′GTTCCAGWACAGSAKRTCGAA3′) by [9] targeting both the class I and class II synthase gene (*phaC*). Class III and IV synthase gene fragments are not prone to amplification by the primer set used. PCR conditions were as follows: 1 cycle of 94 °C for 10 min, 60 °C for 2 min and 72 °C for 2 min followed by 40 cycles at 94 °C for 20 s, 55.5 °C for 45 s and 72 °C for 1 min and a final cycle at 72 °C for 5 min using DreamTaq polymerase (Thermo Scientific-Fermentas). Negative controls were added in each PCR run. Obtained PCR products (~550 bp for the *phaC* gene fragment) were checked on 0.8% agarose gel. Samples containing the desired length product were scored as positive.

### 2.4. Feast-Famine Regime Implementation

Three types of PHA-inducing media (PIM) were introduced to create a feast/famine regime based on lowering the nitrogen to carbon ratio. The basis was a 7-fold diluted (0.5 g/L) R3A medium amended with several carbon sources. PIM1 contained carbon sources that were unrelated to PHA structure: glucose (2.5 g/L), sodium lactate (2.5 g/L), glycerol (2.5 g/L) and sodium acetate (1 g/L). PIM2 contained short-chain fatty acids: sodium butyrate and sodium valerate (3.5 g/L each). PIM3 contained a mix of medium-chain fatty acids derived from the ultrasonic-initiated saponification of coconut oil [17]. Ten grams of coconut oil (generic brand) was amended with 30 mL of 1.5 M NaOH (in excess) in a 50 mL Falcon-type tube, heated in a water bath at 50 °C until the oil completely melted. The ingredients were then shaken for 5 min on a Tornado Vortexer until a homogenous emulsion was achieved. Then the tube was placed in a VWR Ultrasonic Cleaner USC-TH set to 50 °C and sonicated at 40 kH for 45 min to facilitate the saponification reaction. The tube was placed in a thermostat at 37 °C for 6 weeks to allow the saponification reaction to proceed to completion. The saponified oil was dissolved in ddH_2_O, the pH was adjusted to 5.0 with HCl upon which the free fatty acids precipitated. The suspension was sedimented by centrifugation (10 min, 8000 rpm) and washed three times with ddH_2_O to remove the excess alkali and the glycerol by-product. Free fatty acids were air-died and suspended in water at a concentration of 7 g/L with Tween80 (0.1 g/L). The mixture was emulsified by heating to 45 °C and vigorous shaking. All media were adjusted to pH 7.2.

Bacterial cultures on R3A broth were harvested by centrifugation (0.5 mL) in separate Eppendorf tubes, resuspended in 3 mL of each of the PIMs, and incubated at 14 °C for 1 week. Resulting suspensions were subjected to microscopy-based screening.

### 2.5. Nile Red Staining and Microscopy

Bacterial suspensions were washed with a washing buffer (NaCl 9 g/L, methanol 10 g/L, Tween80 0 µg/L, tetrasodium pyrophosphate 2.6 g/L) to remove media components and slightly perforate cell walls for dye penetration. Bacterial cells were resuspended in 0.9% saline and stained with Nile Red in DMSO (80 µg/mL) to give a final concentration of 3.1 µg/mL for 30 min [18]. Stained bacterial suspensions were trapped under a microscope slide and observed under 1000× magnification with green (510–560 nm) and blue light (450–490 nm) excitation on a Nikon E-200 microscope with a 100 W Hg lamp and 100× CFI 60 oil immersion objective, with a digital DS-Fi3 high-definition color microscope camera equipped with a 5.9 megapixel CMOS image sensor and a filter block of wavelengths: EX 330–380, DM 400, BA 420.

### 2.6. Data Analysis

Calculation charts and graphs were made in Excel (MS Office for Windows). The simple matching coefficient (SMC) was calculated using the following formula: SMC = (M_00_ + M_11_)/(M_00_ + M_01_ + M_10_ + M_11_), where: M_00_—total number of attributes where both have a value of 0, M_11_—total number of attributes where both have a value of 1, M_01_ and M_10_—total number of attributes where one has a value of 1 and the other a value of 0. The 16S rRNA gene fragments were identified using the Blastn algorithm with the “sequences from type material” option, identifying the strain as the closest match to validly describe species. Phylogenetic trees were made using the Mega-X software [19]. Obtained 16S partial sequences were deposited in the GenBank database under the accession numbers: MT585825-MT586024.

## 3. Results

**Figure 1:**
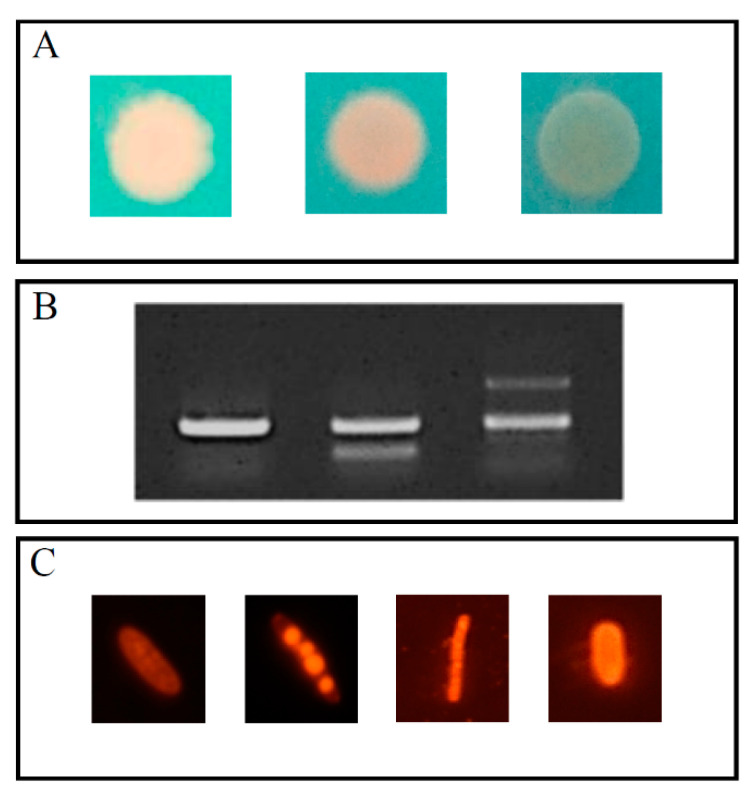
(**A**) Examples of drop growth fluorescence of bacterial strains in UV light on a R3A agar supplemented with Nile Red (from left to right): *Janthinobacterium* sp. 1167, *Chryseobacterium* sp. 966, *Rhodanobacter* sp. 2793. (**B**) Examples of PCR-amplification results (bands) of a *phaC* gene fragment using the G-D and G-1R primers by Romo et al. 2007 (left to right): *Polaromonas* sp. 1701 (550 bp band), *Acidovorax* sp. 1169 (300 bp and 550 bp band), *Janthinobacterium* sp. 1167 (550 bp and 1000 bp band), (**C**) Bacterial cell fluorescence in green light excitation (510–560 nm) after Nile Red staining (left to right): *Janthinobacterium* sp. 1167—“empty” cell, *Janthinobacterium* sp. 1167—granule-filled cell, *Flavobacterium* sp. 1052 cell scored as PHA negative (no granule outline), *Psychromonas* sp. 1212 cell scored as PHA negative (no granule outline).

Polar region strains screened with the Nile Red-amended agar technique displayed varying shades of the expected bright orange fluorescence. Some strains displayed a fluorescence with a pinkish hue (like *Polaromonas* sp. 1701), some with a more yellowish hue (like *Chrysoebacterium* sp. 966). In others, like the *Rhodanobacter* sp. 2793, only a faint trace of the orange fluorescence could be observed at the rim of the drop-growth (Figure 1A). Several strains displayed growth retardation on the Nile Red amended agar in comparison to the Nile Red free control. Agar with the Nile Blue A dye did not yield any specific fluorescence. Amplification of the *phaC* gene resulted in the single, specific 550 bp long fragment like in the case of *Polaromonas* sp. 1701 or in multiple fragments, including the specific one (Figure 1B). Microscopic observations of the Nile Red–stained cells observed under green light excitation in most cases confirmed the orange-fluorescing intracellular granules with a clearly defined morphology thereof. The outline of cells without PHA granules was also visible. Most strains belonging to the Bacteroidetes phylum, like *Flavobacterium* sp. 1052 and also marine bacteria (*inter alia Psychromonas* sp. 1212) displayed an orange fluorescence of the whole cell, without any granule-characteristic morphology (Figure 1C).

**Figure 2:**
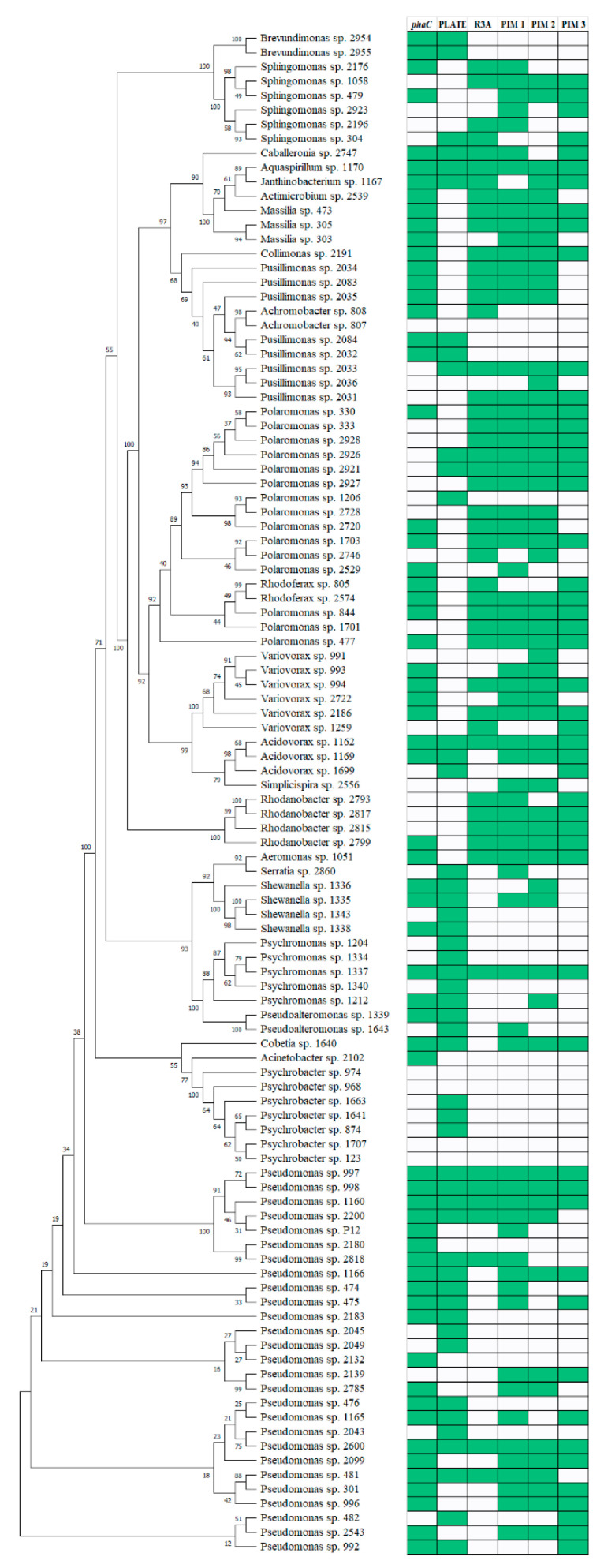
Phylogenetic tree based on partial 16S rRNA gene sequences belonging to the Proteobacteria phylum lined up with the results from the PHA screening with various methods. The tree was built using the neighbor-joining method. Bootstrap values are indicated at the nodes. Green boxes indicate a positive score. *phaC*—presence of the 550 bp DNA fragment of the *phaC* gene; PLATE—presence of characteristic fluorescence on R3A agar plates with Nile Red after UV exposure; R3A—presence of red-fluorescing granules in Nile Red stained cells cultured on R3A broth; PIM1—presence of red-fluorescing granules in Nile Red stained cells incubated in PIM1 medium; PIM2—presence of red-fluorescing granules in Nile Red stained cells incubated in PIM2 medium; PIM3—presence of red-fluorescing granules in Nile Red stained cells incubated in PIM3 medium.

**Figure 3:**
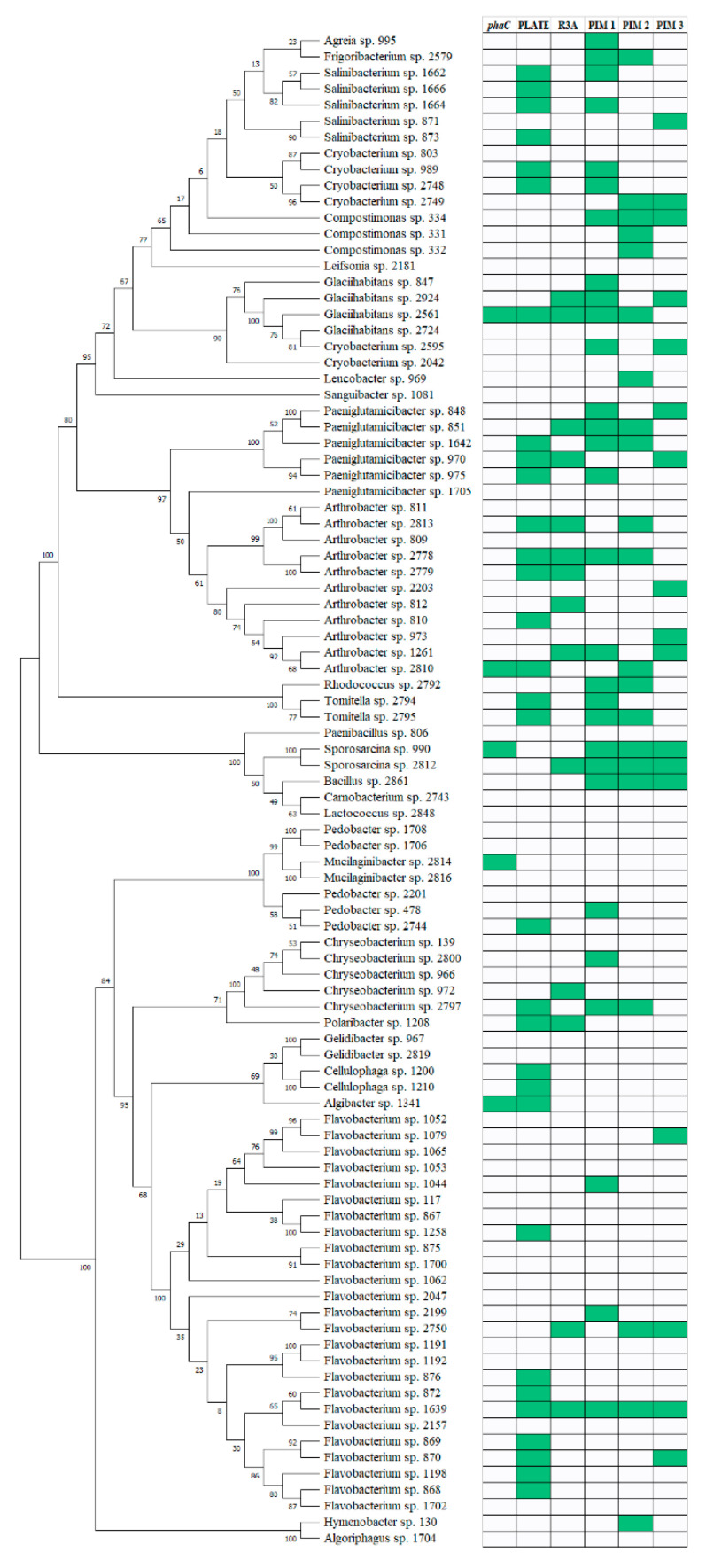
Phylogenetic tree based on partial 16S rRNA gene sequences belonging to the Actinobacteria, Firmicutes, and Bacteroidetes phylum lined up with the results from the PHA screening with various methods. The tree was built using the neighbor-joining method. Bootstrap values are indicated at the nodes. Green boxes indicate a positive score. *phaC*—presence of the 550 bp DNA fragment of the *phaC* gene; PLATE—presence of characteristic fluorescence on R3A agar plates with Nile Red after UV exposure; R3A—presence of red-fluorescing granules in Nile Red stained cells cultured on R3A broth; PIM1—presence of red-fluorescing granules in Nile Red stained cells incubated in PIM1 medium; PIM2—presence of red-fluorescing granules in Nile Red stained cells incubated in PIM2 medium; PIM3—presence of red-fluorescing granules in Nile Red stained cells incubated in PIM3 medium.

Examined isolates belonged to four phyla: Proteobacteria (Figure 2), Actinobacteria, Firmicutes, and Bacteroidetes (Figure 3). Proteobacterial isolates belonged to three classes: Alphaproteobacteria (*n* = 8) consisting mostly of *Sphingomonas* sp. strains. Betaproteobacteria were more numerous consisting of 45 isolates clustered within roughly three families, the most important being the *Comamonadaceae* (*n* = 27), with *Polaromonas* as the most frequent genus. Gammaproteobacterial isolates were the most numerous (*n* = 53) and most diverse (>7 families). The family with the most members was *Pseudomonadaceae* (*n* = 27) consisting exclusively of the *Pseudomonas* genus (Figure 2). Actinobacteria (*n* = 43), Firmicutes (*n* = 6) and Bacteroidetes (*n* = 45) were less frequently isolated than Proteobacteria. Actinobacterial strains belonged mostly to two families: *Microbacteriaceae* (*n* = 22) and *Micrococcaceae* (*n* = 17). *Microbacteriaceae* consisted mostly of three genera: *Salinibacterium*, *Cryobacterium*, and *Glaciihabitans*. The *Micrococcaceae* consisted exclusively of two genera: *Arthrobacter* and *Paeniglutamicibacter*. The Bacteroidetes isolates were mostly members of the *Flavobacteriaceae* family (*n* = 36).

**Figure 4:**
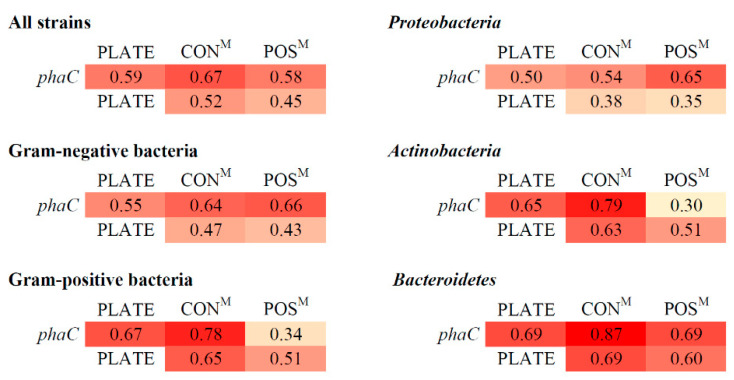
Simple matching coefficient calculated from the binary (positive/negative) data obtained from each screening method for the following subsets: all strains (*n* = 200), Gram-negative bacteria (*n* = 151), Gram-positive bacteria (*n* = 49), Proteobacteria (*n* = 106), Actinobacteria (*n* = 43), Bacteroidetes (*n* = 45). *phaC*—binary dataset of the presence/absence of the 550 bp DNA fragment of the *phaC* gene; PLATE—binary dataset of the presence/absence of the characteristic fluorescence on R3A agar plates with Nile Red after UV exposure; CON^M^—microscopy-obtained (M) binary dataset of the presence/absence of granules after incubation in R3A broth (constitutive producers); POS^M^—microscopy-obtained (M) binary dataset of the presence/absence of granules after incubation on ≥1 of the liquid media used (R3A, PIM1,2,3).

To examine the reproducibility of the results obtained by the different methods a simple matching coefficient (SMC) was calculated between each of the binary data sets, taking both negative and positive responses into account (Figure 4). The SMC results vary between one and zero, where one means that data sets are identical and zero if there are no identical scores within the two datasets. As the microscopy-based method was evaluated as the least bias-prone, strains were included in the PHA-positive group (POS) when granules were present after cultivation on either of the liquid media used (R3A, PIM1,2,3). None of the methods used showed a total result overlap. For the “all strains” group, the highest overlap was achieved for the *phaC* gene detection and microscopy method for R3A medium cultured strains (CON—constitutive PHA producers)—0.67. Low overlap values were achieved for the agar plate screening method (0.45–0.59). For Gram-negative bacteria, this was also apparent with high overlap of the *phaC* detection with strains scored as positive (0.66). Gram-positive bacteria on the other hand had a low overlap (0.34) of the *phaC* detection scores with positive strains but high overlap with a microscopy-based method for constitutive producers. The plate method displayed in the case of Gram-positive bacteria greater overlap with other methods (0.65–0.67). Proteobacteria displayed low to moderate overlap values, especially low for the agar plate method (0.35–0.50). The highest overlap value was achieved for the *phaC* detection and PHA positives. Actinobacteria displayed a relatively high overlap between *phaC* detection and microscopy method for constitutive producers (0.78) and low overlap when the overlap was calculated for PHA positive strains. Bacteroidetes displayed moderate to high overlap of tested methods with the highest between *phaC* detection and microscopy granule detection after cultivation on R3A broth (Figure 4).

**Figure 5:**
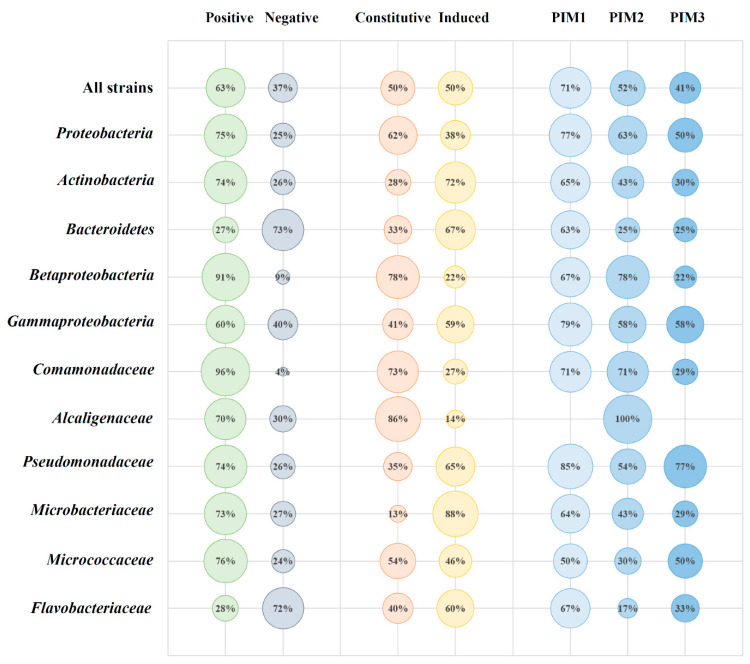
Percentage of strains (total and within selected taxonomic ranks) expressing the following traits: Positive—presence of red-fluorescing granules (on R3A broth and on PIMs); negative—absence of red-fluorescing granules in any conditions; constitutive—presence of red-fluorescing granules in R3A broth; induced-presence of red-fluorescing granules only after incubation in PIMs; PIM1—presence of red-fluorescing granules after incubation in PIM1 within the “Induced” group; PIM2—presence of red-fluorescing granules after incubation in PIM2 within the “Induced” group; PIM3—presence of red-fluorescing granules after incubation in PIM3 within the “Induced” group.

The percentage of PHA positive strains were based on microscopy observations (Figure 5). For all examined strains, a value of 63% of PHA-granule positive was achieved. Within the phylum-rank group, high values were scored for Proteobacteria (75%) and Actinobacteria (74%). Bacteroidetes scored only 27% PHA-positive strains. Betaproteobacteria and Gammaproteobacteria scored 91% and 60% positive strains, respectively. Within the family level groups (where *n* > 10) the *Comamonadaceae* displayed the highest values (96%) whereas the *Flavobacteriaceae* displayed the lowest (28%). The *Alcaligenaceae*, *Pseudomonadaceae*, *Microbacteriaceae*, and *Micrococcaceae* remained within a 70–80% threshold (Figure 5). Half of the PHA-producers (within all examined strains) were constitutive granule producers, whereas the other half had to be induced with a high C/N ratio. The highest constitutive producer percentages were found belonging to the *Alcaligenaceae* and *Comamonadaceae* families within the Betaproteobacteria class (86% and 73%, respectively), whereas the *Microbacteriaceae* (Actinobacteria) and *Pseudomonadaceae* (Gammaproteobacteria) displayed a high percentage of strains that produced PHA only upon induction. The most successful induction medium was the PIM1, inducing PHA-formation in 71% of all of the non-constitutive PHA producers. It was the highest-scoring induction medium for all groups except for the Betaproteobacteria and *Alcaligenaceae*, where PIM2 was more successful in inducing PHA formation. PIM3 medium containing medium-length fatty acid salts was the most successful in PHA induction of the *Pseudomonadaceae* family members.

**Figure 6:**
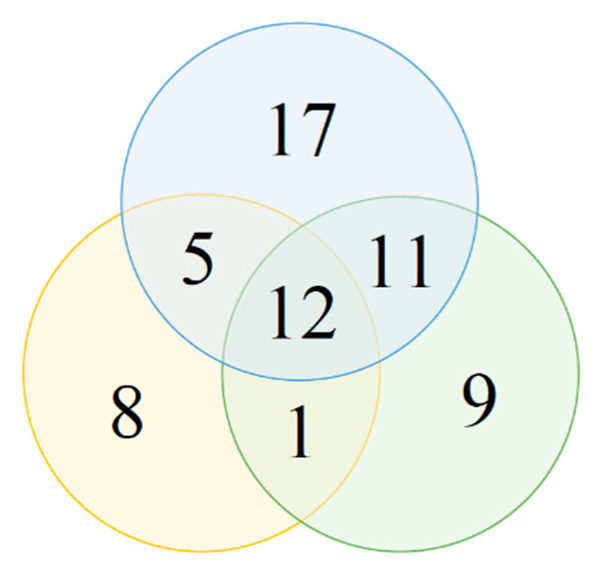
Venn diagram displaying numbers of strains within the “induced” group that were PHA positive on different versions of PIM. Blue circle—PIM1, green circle—PIM2, orange circle—PIM3.

The inducible PHA-granule accumulating strains usually produced the storage material on more than one type of induction media (Figure 6). PIM1 and PIM2 shared the greatest number of induced strains (23) whereas PIM2 and PIM3 shared the lowest number (13). Twelve strains managed to produce PHA on all three induction media.

**Figure 7:**
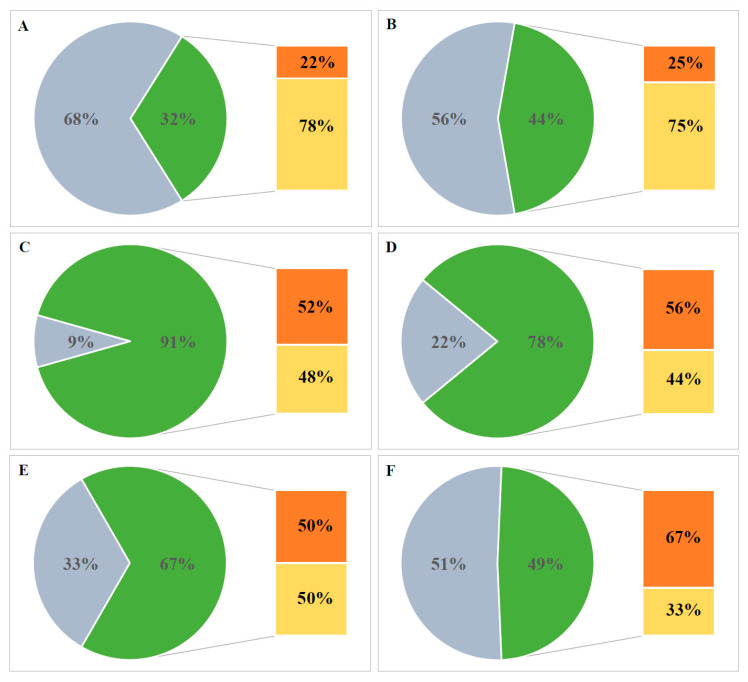
Percentage of strains divided according to habitat-type of origin: (**A**) zoogenic habitat, (**B**) marine habitat, (**C**) glacial habitats, (**D**) pedogenic habitats, (**E**) postglacial habitats, (**F**) freshwater habitats. Percentage of strains positive for granule presence—green part of diagram, negative—gray, constitutive PHA producers within the positives—orange, PIM-inducible PHA producers within the positives—yellow.

Strains were grouped according to the type of environment they were isolated from (Figure 7). Glacial environments harbored the most PHA-positive strains (91%), 52% of which were constitutive granule accumulators. High contribution values of PHA positive strains were also apparent for soil-associated (pedogenic) habitats (78%) but also post-glacial deposits (67%). Marine and animal-influenced habitats contained mostly non-PHA producing strains, albeit the PHA producing minority had to be induced to accumulate PHA (>70%). Almost half of the freshwater habitat-isolated strains had PHA synthesis abilities, 67% of which did it without the high carbon to nitrogen ratio induction.

## 4. Discussion

### 4.1. Evaluating Screening Methods

Popular screening methods (both physiology- and gene-based) displayed a varying degree of accuracy when dealing with polar region-derived bacterial strains. The plate method yielded results that were hard to evaluate. Most difficult to interpret were pigmented strains, which was noted also by other researchers [20]. In the case of *Rhodanobacter* sp. isolates the presence of the pigment seemed to extinguish the Nile Red-stained granule fluorescence. *Rhodanobacter* isolates often produce xanthomonadin-like pigments, which absorb wavelengths in the blue-green range, those that excite the granule-bound Nile Red dye [18,21,22]. Drop-growth of most Bacteroidetes members (mostly *Flavobacterium* spp. and *Chryseobacterium* spp. isolates) and several Proteobacteria of marine origin (e.g., *Psychromonas* sp.) displayed strong orange fluorescence on the Nile Red amended agar. This was checked by microscopy analysis, where whole cells had an orange glow in green light excitation. Flavobacteria of polar origin produce substantial amounts of branched and unsaturated fatty acids to maintain membrane fluidity [23], whereas marine psychrophiles produce polyunsaturated fatty acids for the same purpose [24]. Ref. [25] states that those fatty acids belong to the polar lipid group and their fluorescence after Nile Red staining is in the range of 610 nm (orange). A feature of the plate based-screening, besides color evaluation difficulty, was also visible growth retardation on the Nile Red amended agar in comparison to the Nile Red-free control. Presumably, strains that originate from polar regions, where there is low anthropogenic impact and low nutrient concentrations, are more sensitive to xenobiotics, like the diluent used (DMSO) or the dye itself [7,26]. Surprisingly, agar amended with the Nile Blue A dye did not yield any specific fluorescence. Presumably, the low temperature or the pH of the medium hampered its oxidation to the active derivative (Nile Red) [27]. Genetic screening methods have an advantage over the metabolism-based ones as they do not rely on granule presence; however, genetic screening methods can only indicate that strains have the genetic potential to produce PHA but not that the gene is expressed or that PHA is accumulated. The primer set designed by [9] targets type I and II synthase genes. Amplification of the *phaC* fragment using those primers frequently yielded multiple bands with or without the 550 bp specific band during the screening of polar region bacteria. This could be explained either by non-specific binding of the degenerate primers or the existence of other copies of the gene in close proximity to each other, simultaneous carrying of two types of synthases or other unusual PHA synthase related genes. Recent studies [28] have shown, that *Pseudomonas* spp. isolated from Antarctica carried two classes of synthases (I and II) and some *Janthinobacterium* sp. isolates contained genes of a usual PHA synthase (provisionally named class V) besides the class I synthase. Class III and IV synthase genes were not amplified by the primers used and currently, there are no wide-range primer sets to detect them [29]. Microscopy observation of the Nile Red stained cells also required cautious examination. However, the microscopy-based approach has proven the most accurate and the most reliable down the line, as it detected active PHA accumulators and granule presence was not only confirmed by the characteristic fluorescence but also by their morphology [30]. As already mentioned, marine strains and those belonging to the Bacteroidetes phylum displayed a whole-cell red fluorescence in green light excitation. Those were scored as negative for PHA accumulation as the granules in native producers tend not to exceed 40% of the cells volume [31]. A further advantage of the microscopy-based approach was that the cells were stained *post*-growth and PHA production so there were no issues with a proliferation-hampering effect of the dye or the carrier diluent (DMSO). Furthermore, the feast-famine regime could easily be incorporated into the method to further increase the number of PHA-positive strains.

Comparison of the data with the simple matching coefficient clearly showed that each method produced different results for the same sample group. Assuming that the microscopic method is the most accurate one and considering the results of the feast/famine regime, several observations can be made. The plate-based method displayed low overlap with the microscopy method in Proteobacteria, suggesting that in this group the agar plate approach potentially introduces a heavy bias. In Actinobacteria, *phaC* detection method showed high overlap with the microscopy-based technique only for constitutive producers and low overlap when also inducible producers were taken into account. This points towards the failed amplification of most of the actinobacterial *phaC* gene fragments. The reason being, that the primers were designed mostly on the basis of proteobacterial sequences or that polar region actinobacterial genes represent a novel quality among PHA synthases.

### 4.2. Biodiversity of Psychrophilic PHA Producers

The most frequently isolated polar region PHA-producing strains belonged mostly to the *Comamonadaceae* family. Members of this family have been frequently recognized as a vital part of polyhydroxybutyrate-producing consortia enriched from a mixed microbial culture under different feeding conditions [32,33]. Furthermore, members of the *Comamonadaceae* like *Rhodoferax* spp. and *Polaromonas* spp. have been found to produce PHA in sea ice and cold waters of the northern Baltic Sea [34]. *Polaromonas* spp. are frequent residents of glacial Arctic and Antarctic habitats and are suspected to be involved in symbiosis with eukaryotic algae [35]. This is in line with the conclusions drawn by [36] about the pivotal role of PHA in bacterial symbionts of eukaryotic organisms. Especially surprising is the constitutive feature of the betaproteobacterial PHA synthesis. The need for constant granule presence might be dictated by the sudden changes in nutrient availability or intensity of metabolism-challenging factors as the PHA are thought to protect extremophiles from environmental stressors [15]. Gammaproteobacteria, especially the *Pseudomonadaceae* family members produced PHA mostly under nitrogen limiting conditions. Those bacteria are a known group of opportunitrophs, displaying a large variety of adaptive features like fast growth and a wide range of substrate utilization and lytic enzyme production [37], so PHA production may be an asset only in extremely harsh conditions [38]. Actinobacteria were second in terms of isolate numbers of active PHA producers revealed in this study. A vast majority of actinobacterial strains produced PHA only upon induction with a high C/N ratio, most notably the members of the *Microbacteriaceae* family e.g., *Cryobacterium*. *Micrococcaceae* were more evenly divided among constitutive and inducible PHA producers (e.g., *Arthrobacter* spp.). Information on members of those families as PHA producers is limited, however Actinobacteria from polar regions were described as occupying diverse niches [39], so the scenario presented earlier for *Pseudomonadaceae* might also be applicable. As reported by [40], Actinobacteria tend to produce PHA of unusual chemistry, therefore actinobacterial isolates derived from polar region materials might represent an untapped source of novel, biotechnologically relevant polyhydroxyalkanoates.

### 4.3. PHA Producers and Their Habitats

Polar region habitat types differed quite substantially in cultivable bacterial PHA accumulators’ content. The zoogenic habitat type which was represented by sea birds’ (penguins and little auks) nesting sites and marine habitats represented mainly by decaying seaweed heaps displayed the lowest contribution of PHA producers. Seemingly, polar nutrient-rich substratum [41,42] does not promote species that diverge energy and resources towards carbon-storage material synthesis [43,44]. Furthermore, even among isolates displaying this ability, its expression had to be triggered by nitrogen starvation with simultaneous carbon surplus. Therefore, constant high nutrient availability negatively influences the contribution of PHA producers to the community also in polar regions. Contrasting in this respect were the highly oligotrophic glacial habitats. Vital nutrients, especially nitrogen and phosphorus are scarce in those sites [45,46]. Over 90% contribution of PHA positive isolates among glacier-derived bacteria was therefore not surprising. Presumably, labile carbon surplus is present only during the short summer season [47], therefore PHA production is needed in regard to carbon deficit survival. Among glacier derived PHA positive strains there was an even distribution of constitutive and inducible PHA producers, indicating different mechanisms for coping with extreme glacial conditions. As mentioned before, some bacterial groups might use the granules as an abiotic stress-resistance feature, whereas others might rely on different mechanisms, not related to PHA metabolism like pigment synthesis [48]. Soils of varying complexity (developed and postglacial) shared similar characteristics to each other in terms of percentage of PHA-producing strains and their ability for constitutive or inducible granule accumulation. Heterogeneity of such habitats enables the establishment of a multitude of different niches, even among closely related microorganisms [49], hence, the fairly even distribution between non-producers, constitutive producers, and inducible producers. Polar region freshwater habitats are strongly affected by seasonal changes in nutrient quantity and quality, creating a natural feast/famine regime [50]. Consequently, high altitude Himalayan lakes were recently proclaimed as a “bioplastics reservoir” by [51].

## 5. Conclusions

Polar region bacteria present a novel and potent source of PHA-producing microorganisms. Microscopic observation of Nile Red stained cells amended with a feast/famine regime implementation using different carbon sources was proven as the most reliable screening method. Caution is advised when evaluating members of the Bacteroidetes phylum and polar region marine bacteria as they often give false-positive results in the Nile Red dye involving approach. Members of the *Comamonadaceae* family were the most numerous PHA producers by percentage among the 200 examined strains. Most abundant in cultivable PHA accumulators were glacier associated habitats of both polar regions, followed by well-developed soils and postglacial deposits. Nonetheless, to further expand the topic of polar region PHA producers’ additional investigations are in order: a culture-independent approach of the native source material and an in-depth laboratory analysis of bacterial strains.

## Figures and Tables

**Table 1:** Information on origin and cultivation of strains used in analysis.

Strain Numbers	Material of Isolation	Place of Origin	Environment Type	Maintenance Medium
2043, 2045, 2047, 2049, 2099, 2102, 2132, 2139, 2157	Little auk (Alle alle) guano	Hornsund Fiord, Spitsbergen Island, Arctic	Zoogenic	R3A agar
966, 967, 968, 969, 970, 972, 973, 974, 975	Adélie penguin guano	Point Thomas Rookery, King George Island, Antarctica	Zoogenic	R3A agar
1198, 1200, 1204, 1206, 1208, 1210, 1212, 1334, 1335, 1336, 1337, 1338, 1339, 1340, 1341, 1343, 2848	Decaying seaweeds	Hornsund Fiord, Spitsbergen Island, Arctic	Marine	R3A agar with artificial seawater
1639, 1640, 1641, 1642, 1643, 1662, 1663, 1664, 1666, 2861	Decaying seaweeds	Admiralty Bay shore, King George Island, Antarctica	Marine	R3A agar with artificial seawater
2529, 2539, 2543, 2556, 2561, 2574, 2579, 2595, 2600	Cryoconite	Hans Glacier, Spitsbergen, Arctic	Glacial	R3A agar
2720, 2722, 2724, 2728	Cryoconite	Werenskiold Glacier, Spitsbergen, Arctic	Glacial	R3A agar
301, 303, 304, 305, 330, 331, 332, 333, 334	Glacial surface ice	Ecology Glacier, King George Island, Antarctica	Glacial	R3A agar
473, 474, 475, 476, 477, 478, 479, 481, 482	Glacial surface ice	Baranowski Glacier, King George Island, Antarctica	Glacial	R3A agar
803, 805, 806, 807, 808, 809, 810, 811, 812	Plant-free postglacial soil	Ecology Glacier foreland, King George Island, Antarctica	Glacial	R3A agar
989, 990, 991, 992, 993, 994, 995, 996, 997, 998	Postglacial soil with plant debris	Ecology Glacier foreland, King George Island, Antarctica	Glacial	R3A agar
844, 847, 848, 851	Plant-free postglacial soil	Baranowski Glacier foreland, King George Island, Antarctica	Glacial	R3A agar
117, 123, 130, 139, 1258, 1259, 1261	Plant-free postglacial soil	Windy Glacier foreland, King George Island, Antarctica	Glacial	R3A agar
2176, 2180, 2181, 2183, 2186, 2191, 2196, 2199, 2200, 2201, 2203	Arctic tundra soil with moss/lichen debris	Hornsund Fiord, Spitsbergen Island, Arctic	Pedogenic	R3A agar
1044, 1051, 1052, 1053, 1058, 1062, 1065, 1079, 1081	Kettle lake water	Werenskiold Glacier forefield, Hornsund fiord, Spitsbergen, Arctic	Freshwater	R3A agar
1699, 1700, 1701, 1702, 1703, 1704, 1705, 1706, 1707, 1708, 2031, 2032, 2033, 2034, 2035, 2036, 2042, 2083, 2084, 2954, 2955	Freshwater microbial mat	Jasnorzewski Gardens, King George Island, Antarctica	Freshwater	R3A agar
867, 868, 869, 870, 871, 872, 873, 874, 875, 876	Air	Point Thomas Rookery, King George Island, Antarctica	Zoogenic	R3A agar
1160, 1162, 1165, 1166, 1167, 1169, 1170, 1191, 1192	Subglacial water	Subglacial stream, Hans Glacier, Spitsbergen, Arctic	Glacial	R3A agar
P12, 2778, 2779, 2785	*Deschampsia antarctica* rhizosphere soil	Arctowski Station vicinity, King George Island, Antarctica	Pedogenic	R3A agar
2792, 2793, 2794, 2795, 2797, 2799, 2800, 2860	Ornithogenic soil	Arctowski Station vicinity, King George Island, Antarctica	Pedogenic	R3A agar
2810, 2812, 2813, 2814, 2815, 2816, 2817, 2818, 2819	Moss rhizosphere	Arctowski Station vicinity, King George Island, Antarctica	Pedogenic	R3A agar
2921, 2923, 2924, 2926, 2927, 2928	Supraglacial water	Supraglacial stream, Ecology Glacier, King George Island, Antarctica	Glacial	R3A agar
2743, 2744, 2746, 2747, 2748, 2749, 2750	River water	Ariedalen stream, Spitsbergen, Arctic	Freshwater	R3A agar
